# Staged Bi-compartmental Knee Arthroplasty for Contralateral Compartment Failure After Medial Unicompartmental Knee Arthroplasty in Dialysis Patients: Two Case Reports

**DOI:** 10.7759/cureus.62892

**Published:** 2024-06-22

**Authors:** Atsuo Inoue, Yuji Arai, Yasushi Yoshihara, Shuji Nakagawa, Kenji Takahashi

**Affiliations:** 1 Orthopedics, Kyoto Prefectural University of Medicine, Kyoto, JPN; 2 Orthopedic Surgery, Japanese Red Cross Kyoto Daiichi Hospital, Kyoto, JPN; 3 Sports and Para-Sports Medicine, Kyoto Prefectural University of Medicine, Kyoto, JPN

**Keywords:** knee osteoarthritis/ koa, staged bi-uka, contralateral compartmental failure, subchondral insufficiency fracture of the knee, dialysis patients, uni-compartmental knee replacement

## Abstract

Unicompartmental knee arthroplasty (UKA) is a minimally invasive surgical technique with good clinical outcomes; however, its outcomes in patients undergoing hemodialysis are unknown. Herein, we report two cases of patients undergoing hemodialysis who underwent staged bi-compartmental UKA (Bi-UKA) for early contralateral compartment failure after medial UKA. We describe the case of early contralateral compartment failure after medial UKA in two women patients aged 71 and 72 years with a dialysis history of seven and 22 years, respectively. Three months after right medial UKA, she had persistent joint edema and arthralgia after minor trauma, with recurrent gait disturbance in the first case. An MRI showed a bone marrow lesion in the contralateral compartment, and a lateral UKA was added. In the second case, the knee pain worsened without any trigger three years after leaving the medial UKA. A subchondral insufficiency fracture (SIF) was diagnosed by a plain radiograph showing a radiolucent area on the lateral femoral condyle. Gait disturbance did not improve, and a lateral UKA was performed. In our hospital, medial UKA was performed on seven knees of dialysis patients in 10 years since 2011, and contralateral compartment failure was observed in two knees at an early stage. In both cases, lumbar bone density was normal and there was no postoperative overcorrection in leg alignment, but a SIF of the contralateral side occurred, suggesting that bone fragility of the contralateral compartment due to long-term dialysis was the underlying cause. Staged Bi-UKA was minimally invasive and useful as a revision surgery.

## Introduction

The 2021 statistics of the Japanese Society for Dialysis Therapy revealed that the average age of patients undergoing dialysis is 69.67 years, and the percentage of patients aged ≥65 years has been increasing annually, from 42% in 2000 to 69% in 2021 [1]. The number of dialysis patients requiring knee arthroplasty will increase with the increase in knee joint disease owing to the aging population. While total knee arthroplasty (TKA) is a procedure that can be performed for a variety of deformities of the knee, unicompartmental knee arthroplasty (UKA) is now commonly performed in elderly patients with unicompartmental disorders such as medial knee osteoarthritis or femoral medial condyle osteonecrosis. UKA can be expected to have good long-term surgical outcomes, with appropriate surgical indications such as residual anterior cruciate ligament (ACL) and the absence of significant lower extremity alignment abnormalities [2,3]. TKA in hemodialysis patients is advantageous because of its effective pain relief and stable short-term results [4]. However, TKA has associated risks, such as high transfusion rates, deep infections, and high revision rates in the early postoperative period, and should be performed with caution [5-7]. Conversely, although the indications for UKA are more limited compared to TKA, UKA is a minimally invasive surgery with better functional recovery and less risk of blood transfusion and deep infection [8-10]. Therefore, if the main lesion in knee joint disease involves the medial compartment in patients undergoing dialysis, UKA is not contraindicated and may be considered a minimally invasive surgical option. However, the outcomes and complications of UKA in dialysis patients are unknown. To date, medial UKA has been performed on seven knees of dialysis patients since 2011 in our hospital. All dialysis patients were indicated for UKA on preoperative radiographic imaging and MRI. Herein, we report two cases of early contralateral compartment failure after UKA who required additional lateral UKA and state the first results of staged bi-compartmental UKA (Bi-UKA) in dialysis patients.

## Case presentation

Case 1

Preoperative History

**Figure 1:**
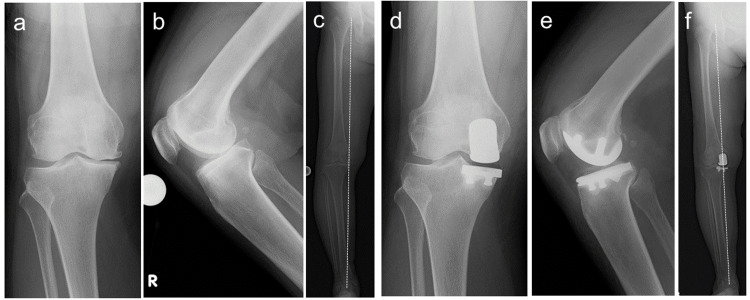
Radiographic images (a-c) Pre-UKA; (d-f) Post-UKA (the broken line indicates the Mikulicz line on the standing radiograph), standing FTA; (c) 185 ° (f) 180 ° FTA, femorotibial angle; UKA, unicompartmental knee arthroplasty

A 71-year-old woman was advised to undergo surgical treatment for persistent right knee joint pain. Renal failure was due to chronic glomerulonephritis, and the patient was on maintenance dialysis. At the time of the initial surgery, the patient had been undergoing dialysis for seven years, and the bone mineral density (BMD) at the lumbar spine was 97% of the Young Adult Mean percentage (%YAM). The preoperative range of motion (ROM) was 0 ° extension and 135 ° flexion, with a preoperative Knee Society Score (KSS) of 40 points. Radiographic imaging revealed osteoarthritic changes in the medial compartment, and the femorotibial angle (FTA) measured in a standing position was 185 ° (Figure 1a-1c). Following right medial UKA (Zimmer-Biomet Persona partial kneeⓇ), better outcomes were reported, with a standing FTA of 180 ° and the KSS improved to 87 points (Figure 1d-1f).

Presenting Symptoms and Imaging Findings

**Figure 2:**
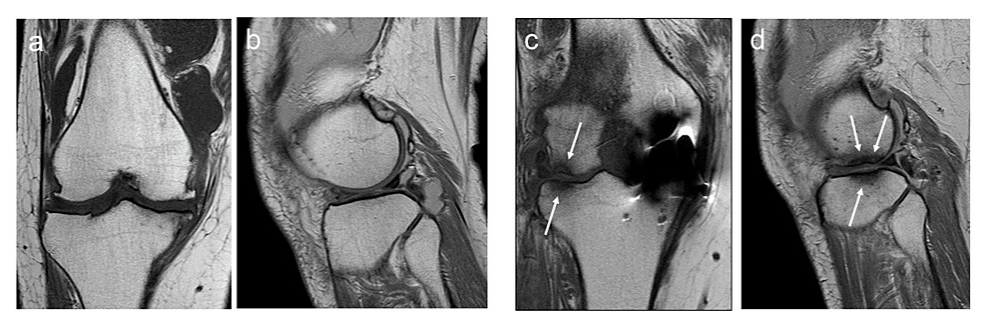
MRI of the (a, b) pre-UKA and the (c, d) post-UKA (a, c) Anterior-posterior images; (b, d) lateral images of the lateral compartment of the knee Arrows indicate bone marrow lesions in the contralateral compartment. UKA, unicompartmental knee arthroplasty

At three months postoperative, the patient stumbled while walking and experienced knee pain. The patient had persistent hydrarthrosis, arthralgia, and recurrent gait disturbances. Although the preoperative MRI showed slight degenerative cartilage change in the lateral compartment of the knee (Figure 2a, 2b), the MRI at four months postoperatively revealed intramedullary changes in the contralateral compartment (Figure 2c, 2d). Arthralgia and hydrarthrosis persisted for five months postoperatively, and the KSS decreased to 62 points.

Staged Bi-UKA and Outcomes

**Figure 3:**
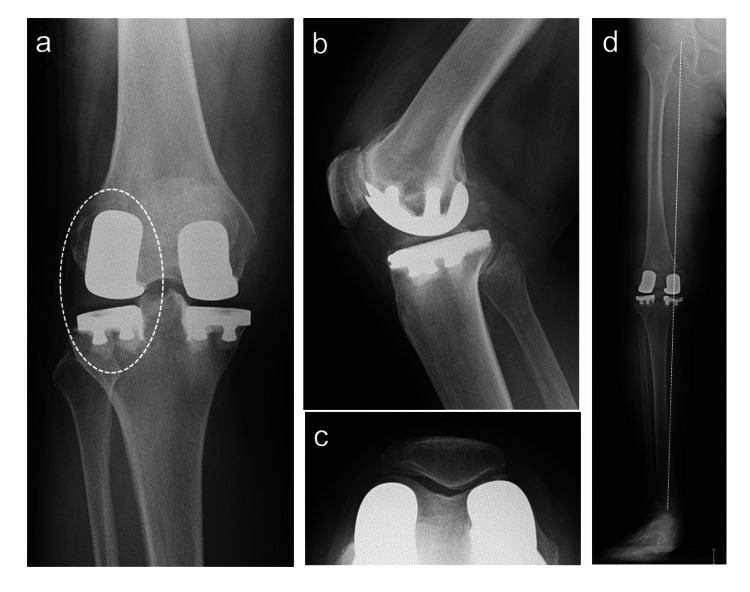
Radiographic images after Bi-UKA (a) Anterior-posterior image (circle indicates the additional lateral UKA); (b) lateral image; (c) axial image of patella; (d) the broken line indicates the Mikulicz line on the standing radiograph; standing FTA is 181 Bi-UKA, bi-compartmental unicompartmental knee arthroplasty; FTA, femorotibial angle; UKA, unicompartmental knee arthroplasty

Because no abnormal findings were observed other than in the lateral compartment, additional lateral UKA was performed with a lateral parapatellar approach. Consequently, the postoperative standing FTA was 181 ° (Figure 3a-3d). Postoperative rehabilitation for Bi-UKA was the same as for medial UKA, with gait training without load restriction. Three weeks after Bi-UKA, the patient was stable when walking with a cane. Six months after Bi-UKA arthralgia and hydrarthrosis resolved, and the KSS improved to 87 points. The patient had a good postoperative course for four years following Bi-UKA, with stable independent walking, 0 ° of extension, and 135 ° of flexion in ROM.

Staged Bi-UKA was minimally invasive and useful as revision surgery for lateral compartment failure after medial UKA in dialysis patients.

Case 2

Preoperative History

**Figure 4:**
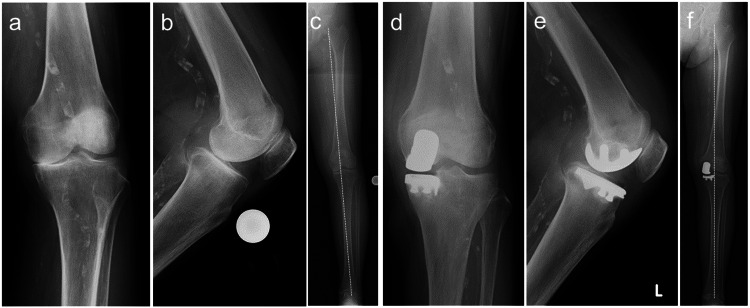
Radiographic images (a-c) Pre-UKA; (d-f) post-UKA (the broken line indicates the Mikulicz line on the standing radiograph), standing FTA; (c) 178 ° (f) 177 ° FTA, femorotibial angle; UKA, unicompartmental knee arthroplasty

A 72-year-old woman with complaints of persistent left knee joint pain for four years was advised to undergo surgical treatment. Relevant clinical history revealed that renal failure was due to acute nephritis, and the patient was undergoing maintenance dialysis. The patient had been on dialysis for 19 years at the time of the initial surgery. Despite a pertinent history of cerebral infarction, no specific sequelae, such as hemiplegia, were present. The preoperative lumbar spine BMD was 83% (%YAM). The preoperative ROM was 0 ° extension and 130 ° flexion, with a preoperative KSS of 55. Medial compartmental osteoarthritis changes were detected on radiographs, with a standing FTA of 178 ° (Figure 4a-4c). Moreover, the MRI showed no obvious abnormality in the ACL; therefore, this was considered an indication of UKA. After the left medial UKA (Zimmer-Biomet Persona partial kneeⓇ), the patient had a good clinical course (postoperative standing FTA 177 °), and the KSS improved to 90 (Figure 4d-4f).

Presenting Symptoms and Imaging Findings

**Figure 5:**
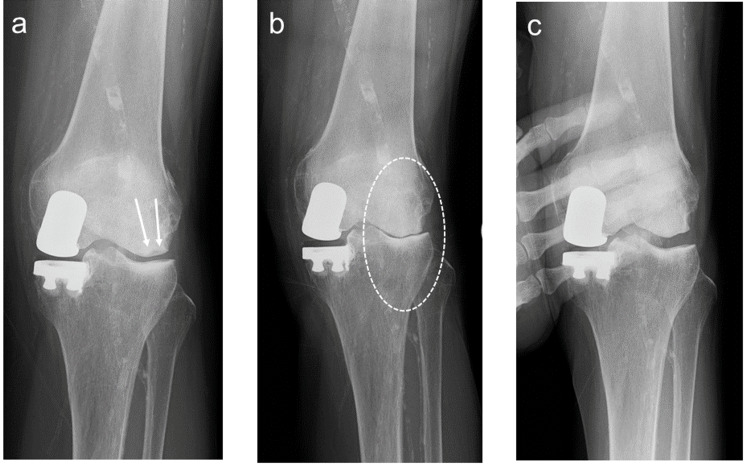
Radiographic images after medial UKA (a) Radiographic image obtained at two years and nine months after UKA (arrows indicate the radiolucent area on the lateral femoral condyle); (b) radiographic image obtained at three years and 10 months after UKA showing narrowing of lateral joint space (the circle indicates the narrowing of the lateral joint space); (c) radiographic image of the knee on varus stress UKA, unicompartmental knee arthroplasty

Two years and seven months postoperatively, the pain in the left knee joint worsened after stamping on the slippery ground, and hydrarthrosis was observed. Because there were no signs of infection or obvious abnormal findings on plain radiographs, the patient was followed up for two months; however, the joint symptoms persisted. Consequently, plain radiography was performed again, which revealed a radiolucent area on the lateral femoral condyle (Figure 5a), and a subchondral insufficiency fracture (SIF) was diagnosed. Owing to the repeated episodes of mild cerebral infarctions, the patient did not wish to undergo surgical treatment, and conservative treatment was continued. No improvement in gait was observed after three years and 10 months, and the ROM of the left knee decreased to 0 ° extension and 120 ° flexion with a KSS of 52 points. The standing FTA was 171 ° and the valgus deformity was progressive (Figure 5b); however, the lower extremity alignment can be corrected with varus stress (Figure 5c).

Staged Bi-UKA and Outcomes

**Figure 6:**
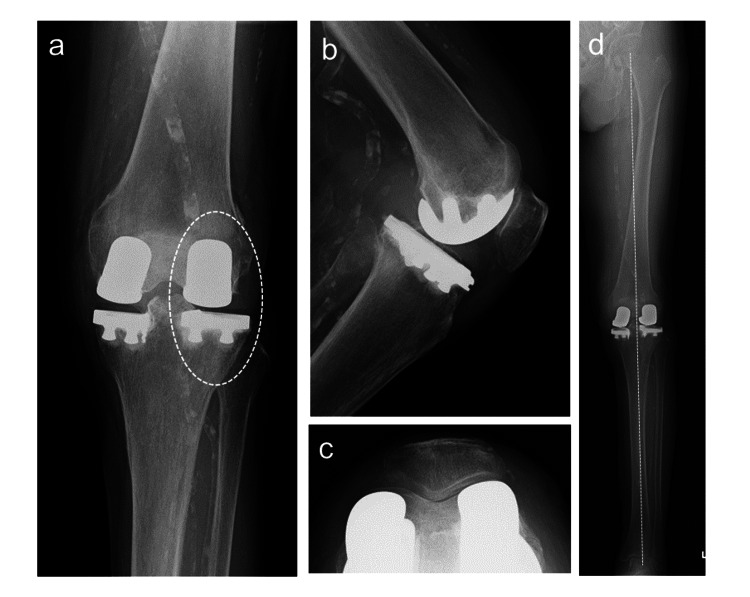
Radiographic images after Bi-UKA (a) Anterior-posterior image (circle indicates the additional lateral UKA); (b) lateral image, (c) axial image of the patella; (d) the broken line indicates the Mikulicz line on the standing radiograph; standing FTA is 175 ° Bi-UKA, bi-compartmental unicompartmental knee arthroplasty

An additional surgery for lateral UKA helped resolve arthralgia (Figure 6a-6c). The additional UKA was performed using the lateral approach. Two years and six months after Bi-UKA, the ROM improved to 0 ° of extension and 130 ° of flexion, with a KSS of 85 points. The standing FTA was 175 ° after Bi-UKA (Figure 6d).

Staged Bi-UKA was minimally invasive and effective even if the deformity progressed after the lateral compartment failure in long-term dialysis patients, as long as the alignment was correctable and there was no loosening in the medial UKA.

## Discussion

Contralateral compartment disorders rarely occur following UKA. Pandit et al. reported 2.5% of 1,000 mobile-bearing UKA cases at 15-year follow-up [11]. Parratte et al. reported that contralateral compartment failure occurred in 1.3% of cases of fixed-bearing-type UKA with an average of 10 years and in 6.5% of cases of mobile-bearing-type UKA with an average of 7.1 years [12]. At our institution, medial UKA has been performed on seven knees of dialysis patients in 10 years since 2011 (Table 1), and contralateral compartment failure was observed in two knees during follow-up (Table 1, #4 and #5). Furthermore, the early failure in these cases, such as at three months or two years and seven months postoperatively, indicates that a high rate of early contralateral compartment failure may occur after UKA in hemodialysis patients. There were no characteristic differences in age, BMI, dialysis history, causes of renal failure, or lower extremity alignment changes between the two patients and the other five patients (Table 1). Further investigation is needed to clarify the factors that led to early failure in these two cases. In terms of pathology, in Case 1, MRI after minor trauma showed low-density areas in the contralateral femoral and tibial sides on proton density-weighted images, leading to a diagnosis of SIF (Figure 2c, 2d). In Case 2, plain radiographs obtained two months after the worsening of symptoms showed a radiolucent area on the lateral femoral condyle, which was diagnosed as SIF (Figure 5a). These findings suggest that fragility fractures of the contralateral compartment in both cases could be attributed to minor trauma in the early postoperative period. Bone quality, as well as BMD, has been reported to deteriorate in hemodialysis patients. Dual-energy X-ray absorptiometry can evaluate the bone quantity but not the bone quality [13]. Both cases did not show a marked decrease in BMD, but there may have been a local loss of bone quality associated with long-term dialysis. The fact that postoperative rehabilitation was similar to the usual UKA may have influenced the contralateral compartment disorders, and in the case of dialysis patients, careful weight-bearing walking may be necessary during postoperative rehabilitation.

**Table 1:** Summary of UKAs of dialysis patients in our institution Case 1 is patient #4, while Case 2 is patient #5. FTA, femorotibial angle; UKA, unicompartmental knee arthroplasty

Patient	Age	Gender	BMI	Duration of dialysis	Causes of renal failure	Pre-FTA/Post-FTA
							#1	74	Male	22.9	1 year	Nephrosclerosis	181°/178°
#2	74	Male	21.7	1 year	Chronic glomerulonephritis	180°/176°
#3	72	Female	23.7	16 years	Diabetic nephropathy	182°/180°
#4	71	Female	27.2	6 years	Chronic glomerulonephritis	185°/180°
#5	72	Female	20.6	19 years	Acute nephritis	178°/177°
#6	73	Female	20.6	20 years	Acute nephritis	177°/173°
#7	70	Female	20.3	2 years	Chronic glomerulonephritis	180°/178°

Overcorrection of the deformation in UKA could be attributed to the contralateral compartment failure after UKA surgery [14]. However, the postoperative standing FTA in cases 1 and 2 was 180° and 178°, respectively, and there was no overcorrection; therefore, the possibility that excessive stress occurred on the contralateral side was minimal. However, lower extremity alignment changes after UKA, as the medial collateral ligament (MCL) laxity associated with lost articular cartilage and deformed articular surfaces is corrected to the proper MCL tension. Owing to the nature of the UKA procedure, the postoperative contralateral compartment pressure is higher than the preoperative pressure. Compared to high tibial osteotomy, which corrects alignment extra-articularly, the postoperative contralateral compartment pressure increases dramatically in UKA, which corrects alignment intra-articularly [15]. In patients not undergoing dialysis, increased pressure may have little or no effect on the contralateral side if alignment correction for arthropathy is adequate in UKA. However, because of the underlying bone fragility [16,17] of the contralateral compartment in dialysis patients due to chronic renal failure and long-term dialysis, the contralateral bone tissue cannot tolerate changes in the contralateral compartment pressure, even with proper alignment correction, as in these cases. On the other hand, since moderate MCL tension is necessary for long-term results in all patients, including dialysis patients, in situ alignment is not recommended. We believe that TKA should be selected for patients with large changes in alignment due to preoperative valgus stress, as they are not indications for UKA.

Although surgical revision with TKA is an option for contralateral compartment failure after UKA, the surgical invasiveness is often greater than that of staged Bi-UKA because the tibial implant of TKA may require metal augmentation or a stem owing to bone loss in the medial tibia after removal of the UKA [18]. In both of these cases presented here, additional lateral UKA was effective because there was no loosening of the medial UKA, the lesion was confined to the lateral compartment, and the lower extremity alignment could be corrected manually. Staged Bi-UKA is a minimally invasive and useful revision surgery for contralateral compartment failure after UKA.

## Conclusions

We reported two cases of patients undergoing hemodialysis who underwent staged Bi-UKA for early contralateral compartment failure after medial UKA. Staged Bi-UKA is a minimally invasive and useful revision surgery; however, the contralateral bone fragility in patients with long-term dialysis should be taken into account when deciding the indication for UKA.
